# A Systematic Pan-Cancer Analysis of CASP3 as a Potential Target for Immunotherapy

**DOI:** 10.3389/fmolb.2022.776808

**Published:** 2022-04-29

**Authors:** Zheng Zhou, Shiying Xu, Liehao Jiang, Zhuo Tan, Jiafeng Wang

**Affiliations:** ^1^ Department of Head and Neck Surgery, Centre of Otolaryngology-head and Neck Surgery, Zhejiang Provincial People’s Hospital, People’s Hospital of Hangzhou Medical College, Hangzhou, China; ^2^ Bengbu Medical College Graduate School, Bengbu, China; ^3^ Zhejiang Provincial Key Laboratory of Diagnosis and Treatment of Endocrine Gland Diseases, Hangzhou, China

**Keywords:** CASP3, cancer, prognosis, immune infiltration, tumor microenvironment

## Abstract

*CASP3* is the gene encoding caspase-3, a specific protease that cleaves substrates such as poly-ADP ribose polymerase and acetyl-DEVD-7-amino-4-methylcoumarin. This enzymatic activity leads to DNA fragmentation, which is a hallmark of apoptosis. Although recent studies have demonstrated that CASP3 plays a vital role in tumour suppression by promoting apoptosis, these reports did not consider systematic pan-cancer analyses. Therefore, we performed a specific pan-cancer analysis using The Cancer Genome Atlas and Genotype-Tissue Expression databases to analyse *CASP3* expression in terms of cancer prognosis, DNA methylation status, tumour mutative burden (TMB), and microsatellite instability (MSI), as well as immune cell infiltration in different tumours and the molecular mechanisms underlying these. We found that *CASP3* expression was significantly associated with the prognosis of most tumours. Additionally, promoter methylation status was associated with *CASP3* expression in bladder urothelial carcinoma, oesophageal carcinoma, kidney renal clear cell carcinoma, kidney renal papillary cell carcinoma, lung squamous cell carcinoma, prostate adenocarcinoma, sarcoma, testicular germ cell tumours, and uterine corpus endometrial carcinoma. TMB and MSI were associated with *CASP3* expression in 15 tumours. Moreover, *CASP3* expression was correlated with the tumour microenvironment in nearly all tumour types. Further, we observed that in addition to apoptosis, *CASP3* action plausibly involves B cell activation, antigen presentation, immune responses, chemokine receptors, and inflammatory function. Our study thus provides a relatively comprehensive understanding of the carcinogenicity of *CASP3* in different tumours and suggests that *CASP3* is a potential prognostic marker.

## Introduction

Epidemiological studies have shown that global cancer incidence is increasing annually, being expected to surpass coronary artery disease as the leading cause of death worldwide by 2060 (Mattiuzzi and Lippi, 2019). In recent years, remarkable progress has been made in tumour immunotherapy, and an increasing number of immunotherapy drugs have been approved for clinical use (Bulk et al., 2018). However, there is no definitive treatment for cancer. With the development of many clinical databases, such as The Cancer Genome Atlas (TCGA), pan-cancer analysis has facilitated exploration of related pathways and molecular mechanisms in specific tumours and the evaluation of their effects on prognosis. Thus, an emerging trend in tumour therapy includes identifying new potential therapeutic targets in addition to traditional surgical therapy (Blum et al., 2018).

Caspase-3 (CASP3) is a key enzyme in the apoptotic pathway that plays an important role in tumorigenesis and cancer progression (Crowley and Waterhouse, 2016). Thus, *CASP3* activation is often used by researchers as an alternative marker to evaluate the efficacy of cancer treatments. However, numerous studies have indicated that *CASP3* does not simply inhibit tumour growth, whereas others have reported that *CASP3* activation after chemical or radiation exposure may be associated to carcinogenesis (Liu et al., 2015). Several studies have shown that *CASP3* promotes tumour growth by creating a microenvironment that promotes angiogenesis (Feng et al., 2017; Bernard et al., 2019). In a colon cancer study, *CASP3* was found to play a role in tumour invasion and metastasis, and the deletion of *CASP3* often indicates higher sensitivity to chemotherapy and radiation, suggesting that cleaved CASP3 may serve as a new therapeutic target in cancer (Zhou et al., 2018).

However, related studies have not sufficiently elucidated the mechanisms of apoptosis, and the specific role of *CASP3* in different tumours remains unclear. Therefore, we used the TCGA, Genotype-Tissue Expression (GTEx), and other databases to conduct a specific pan-cancer analysis of *CASP3*. In addition to *CASP3* expression in different tumours and its prognostic implications, our analysis included gene mutations, methylation, tumour mutational burden (TMB), microsatellite instability (MSI), and potential associations of *CASP3* in 33 tumour types. Regarding the tumour microenvironment, fibroblast and immune cell infiltration, co-expression of *CASP3*, and related pathways were also analysed. Our findings may provide foundation for new strategies for the treatment of related tumours.

## Materials and Methods

### Data Processing and Differential Expression Analysis

Using UCSC Xena (https://Xena.UCSC.edu/), an online tool for exploring gene expression and clinical and phenotypic data, we downloaded the RNA sequence, somatic mutation, and related clinical data from the TCGA (comprising 10,201 samples from 33 types of cancer). Gene expression data from 31 different normal tissues were downloaded from the GTEx database (https://commonfund.nih.gov/GTEx). All gene expression data were normalised using log2 transformation. Normal and cancer tissues were compared using the *t*-test. The Kaplan-Meier curve, logarithmic rank test, and Cox proportional hazard model were used in all survival analyses. The correlation between variables were calculated using the Spearman or Wilcoxon tests. All statistical analyses were performed using R software (version 4.0.2 or 3.6.3, https://www.r-project.org). Statistical significance was set at *p* < 0.05.

### Gene Expression Analysis

We entered “CASP3” in the tumour immune estimation resource (TIMER2) “Gene DE” module (version 2, http://timer.cistrome.org/) to investigate the differences in *CASP3* expression among different tumours or specific tumour subtypes in the TCGA data using tumour and adjacent normal tissues. Certain tumours did not have the corresponding normal tissue samples or displayed lower expression in normal tissues. Under these conditions, we entered “CASP3” in the gene expression profiling interactive analysis (GEPIA2; http://gepia2.cancer-pku.cn/#index), an online tool for gene expression and co-expression analysis, in the “DIY Expression” module’s “Box plot”, with the following parameters: *p*-value cut-off = 0.01, log2FC (fold change) cut-off = 1, log2 (TPM + 1) for log-scale, and “Match TCGA Normal and GTEx”, and obtained the differential scatter plot and the expression bodymap of *CASP3* between tumours and corresponding normal tissues in the GTEx database. The UALCAN portal (http://ualcan.path.uab.edu/analysis-prot.html) is an online tool for the proteomic analysis of several types of cancer. We used the Clinical Proteomic Tumour Analysis Consortium (CPTAC) dataset for protein expression analysis. We analysed the expression of CASP3 in tumour and normal tissues by inputting “CASP3”. Six available tumour datasets were selected, namely breast, ovarian, colon, clear cell renal cell carcinoma (RCC), UCEC, and lung adenocarcinoma (LUAD). Subsequently, to evaluate the difference in CASP3 expression at the protein level, we downloaded and compared the immunohistochemistry (IHC) images for CASP3 protein in normal tissues and six tumour tissues from the Human Protein Atlas (HPA; http://www.proteinatlas.org/).

### Prognostic Survival Analysis

By combining gene expression and clinical data from each sample extracted from the TCGA database, the relationship between the *CASP3* expression and patient prognosis was studied using four indices: overall survival (OS), disease-specific survival (DSS), disease-free interval (DFI), and progression-free interval (PFI). Kaplan-Meier and log-rank tests were used for the survival analysis (*p* < 0.05). The prognostic data were visualised using R software (version 3.6.3), with the R packages “survival” and “survminer”. In addition, the Cox analysis used the R packages “survival” and “forestplot” to determine the pan-cancer relationship between *CASP3* expression and survival. We then obtained the stage differences of CASP3 in different tumours using the GEPIA2 “Stage plot” module and constructed the relevant violin plots using log2[TPM + 1].

### Gene Methylation Analysis

We entered the gene “*CASP3*” in the UALCAN portal and analysed the differences in methylation expression in tumour and normal tissues. Thereafter, we used SurvivalMeth (http://bio-bigdata.hrbmu.edu.cn/survivalmeth/), an online tool for studying the effect of gene methylation on prognosis, with the parameters set as Method “T-test” and “Threshold Value” = 0.01, the “Maxstat” grouping strategy, and the remaining settings at “Without Restriction” to obtain the effect of *CASP3* methylation levels on overall survival in TCGA database and the Kaplan-Meier survival curve with a statistically significant *p*-value (*p* < 0.05).

### Gene Mutation Analysis

On the cBioPortal website (https://www.cbioportal.org/), an interactive exploration dataset for multiple cancer genomics, we selected “TCGA PanCancer Atlas study” in the “Quick By Gene” and entered “CASP3” to query for genetic mutation-related characteristics of *CASP3*. Mutation-related results were observed in all the TCGA tumours in the “Cancer Type Summary” module. Information regarding the *CASP3* mutation site can be displayed in a protein structure sketch map or three-dimensional (3D) structure using the “Mutation” module. We also used the “Comparison/Survival” module to obtain OS, DSS, DFI, and PFI data for TCGA cancer cases with more *CASP3* mutations and generated the Kaplan-Meier survival curve. TMB is defined as the total number of somatic gene coding, base insertion, replacement, or deletion errors detected per million bases (Yarchoan et al., 2017), which is an important biological indicator of the extent of mutation in tumours; a higher TMB often indicates better outcomes for tumour immunotherapy (Le et al., 2015). The MSI results were obtained as a result of functional defects in DNA mismatch repair in tumour tissues, and MSI-H tumours often imply better treatment outcomes (Lin et al., 2020). We then analysed the association between *CASP3* expression and TMB using mutation data from the TCGA database, derived from the 2018 study by Thorsson *et al.* (Thorsson et al., 2018) using R software (version 4.0.3); *p* < 0.05 was considered statistically significant. We simultaneously analysed the correlation between *CASP3* expression and MSI data obtained from the study by Bonneville *et al.* (Bonneville et al., 2017).

### Tumour Microenvironment Analysis

Before analysing the tumour microenvironment, we first analysed the correlation between the expression of *CASP3* and eight immunologic checkpoints (*SIGLEC15*, *ID O 1*, *CD274*, *Havcr2*, *PDCD1*, *CTLA4*, *LAG3*, and *PDCD1LG2*). The expression values of these eight genes and *CASP3* in all tumours were extracted, and a correlation heatmap was visualised using R software (version 4.0.3). We used “the cancer-associated fibroblast” module in TIMER2 to explore the relationship between *CASP3* expression and fibroblast infiltration, and used the EPIC, MCP-counter, and TIDE algorithms to evaluate the data, which were visualised as a heatmap and scatter plot. Thereafter, we used the “Outcome” module of TIMER2 to analyse the prognosis and obtain the Kaplan-Meier survival curve with *p* < 0.05. We then used the previously downloaded mRNA data from TCGA database and the latest algorithms from TIMER, xCell, MCP-counter, CIBERSORT, EPIC, and quanTIseq with the R package “Immunedeconv” (R software, version 4.0.3) (Sturm et al., 2020) to perform immune infiltration analysis and evaluate the correlation between *CASP3* expression and infiltration of immune cells in different tumours, which was then visualised as a heatmap. The Kaplan-Meier curve was obtained using the same methods.

### Tumour Enrichment Analysis

Gene Ontology (GO) and Kyoto Encyclopaedia of Genes and Genomes (KEGG) gene sets were downloaded from the GSEA website (https://www.GSEA-msigdb.org/GSEA/downloads.jsp). Functional analysis was performed using the R software (version 3.6.3) packages “Limma”, “Org.Hs.eg.db”, “DOSE”, “ClusterProfiler”, and “Enrich plot” to visualise the five most significant *CASP3* enrichment pathways in different tumours. *p* < 0.05 was considered statistically significant.

### *CASP3*-Related Gene Enrichment Analysis

We used the gene name “CASP3” and the “*Homo sapiens*” condition to search the STRING database (https://string-db.org/) with the parameter minimum required interaction score (“Low confidence (0.150)”), meaning of network edges (“evidence”), and max number of interactors (“no more than 50 interactors” in 1st shell), to obtain the CASP3-associated protein network map. Next, we used the GEPIA2 “Similar gene detection” module based on TCGA and GTEx datasets to obtain the first 100 genes closely related to *CASP3*. In addition, the co-expression of *CASP3* and related genes in different tumours was plotted using the R package “Limma” (R software, version 3.6.3). Thereafter, *CASP3* and the selected genes, namely *DDX46*, *GNAI3*, *PDS5A*, *SCYL2*, and *TMPO*, were analysed using “Correlation analysis” in GEPIA2 and a scatter plot was obtained. We used two sets of data to perform the GO and KEGG enrichment analysis and used R software (version 4.0.3) and the packages “ClusterProfiler”, “Org.Hs.eg.db”, “Enrichplot”, and “ggplot2” to visualise the data as bubble plots. The first five correlation pathways were selected to draw the loop plot of the related genes.

## Results

### Gene Expression Analysis Data

**FIGURE 1:**
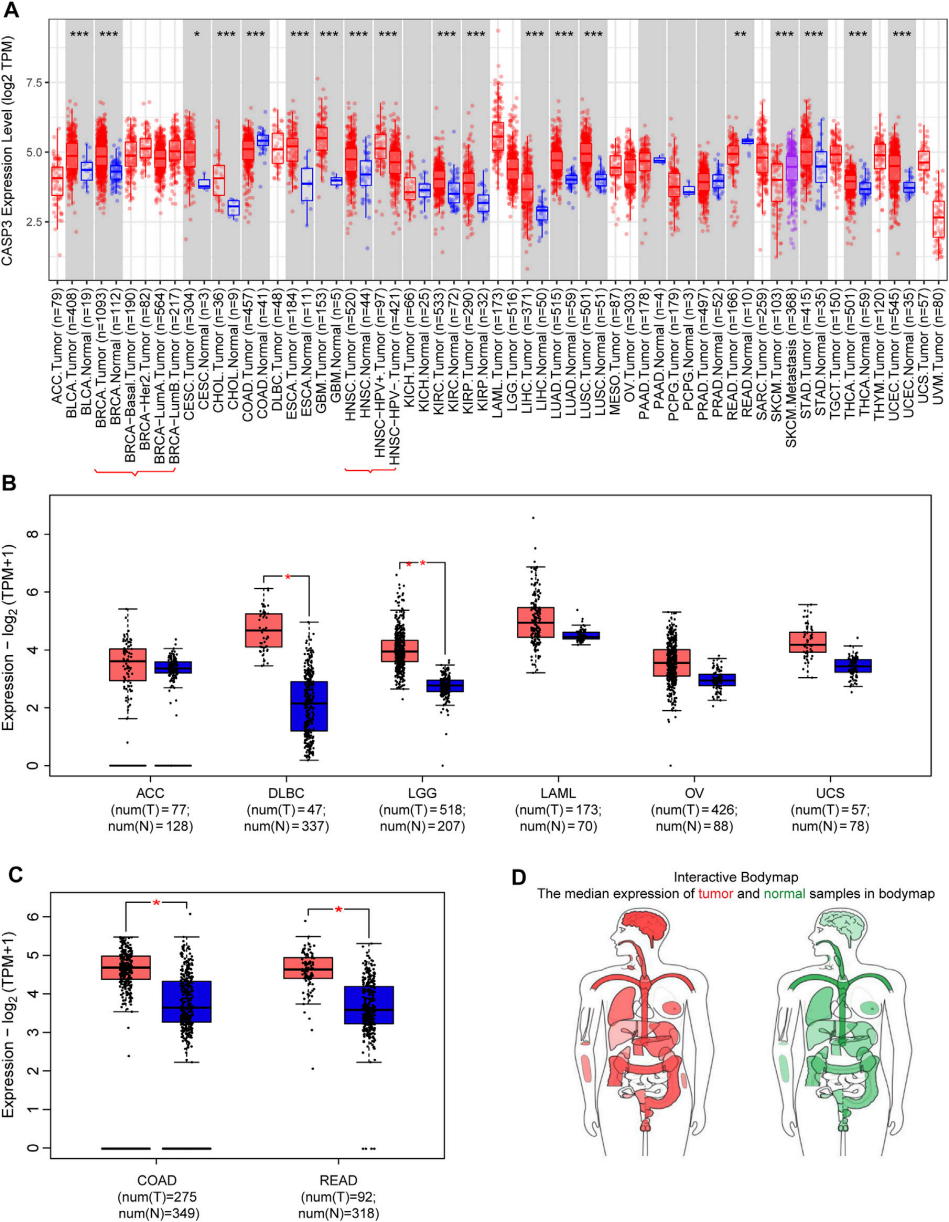
Expression of *CASP3* gene in different tumours. **(A)** Analysis of the expression of *CASP3* in different tumours or specific cancer subtypes by TIMER2. *p < 0.05, **p < 0.01, ***p < 0.001. **(B,C)** For ACC, DLBC, LGG, LAML, OV, uterine carcinosarcoma (UCS), COAD and READ in the TCGA database, the corresponding normal tissues in the GTEx database are used as controls and provided boxplot data. *p < 0.05. **(D)** map of *CASP3* expression in tumours in different parts of the human body by GEPIA2.

We first analysed the differential expression of *CASP3* among different tumours in the TCGA database using the TIMER2 method (Figure 1A) We observed a statistically significant overexpression in bladder urothelial carcinoma (BLCA), breast invasive carcinoma (BRCA), cervical squamous cell carcinoma and endocervical adenocarcinoma (CESC), cholangiocarcinoma (CHOL), oesophageal carcinoma (ESCA), kidney renal clear cell carcinoma (KIRC), kidney renal papillary cell carcinoma (KIRP), lung squamous cell carcinoma (LUSC), glioblastoma multiforme (GBM), head and neck squamous cell carcinoma (HNSC), liver hepatocellular carcinoma (LIHC), lung adenocarcinoma (LUAD), stomach adenocarcinoma (STAD), thyroid carcinoma (THCA), and uterine corpus endometrial carcinoma (UCEC). For tumours with no or few corresponding normal tissue samples, such as lymphoid neoplasm, diffuse large B-cell lymphoma (DLBC), brain lower grade glioma (LGG), *CASP3* as a negative expression of colon adenocarcinoma (COAD), and rectum adenocarcinoma (READ), we used normal tissue expression from the GTEx database added to GEPIA2 to analyse the results (Figures 1B,C). In previously negative and non-normal tumours, *CASP3* expression was significantly higher in DLBC, LGG, COAD, and READ tissues compared with that in normal tissues (*p* < 0.001). *CASP3* expression was high in almost all other human tumours (Figure 1D).

**FIGURE 2:**
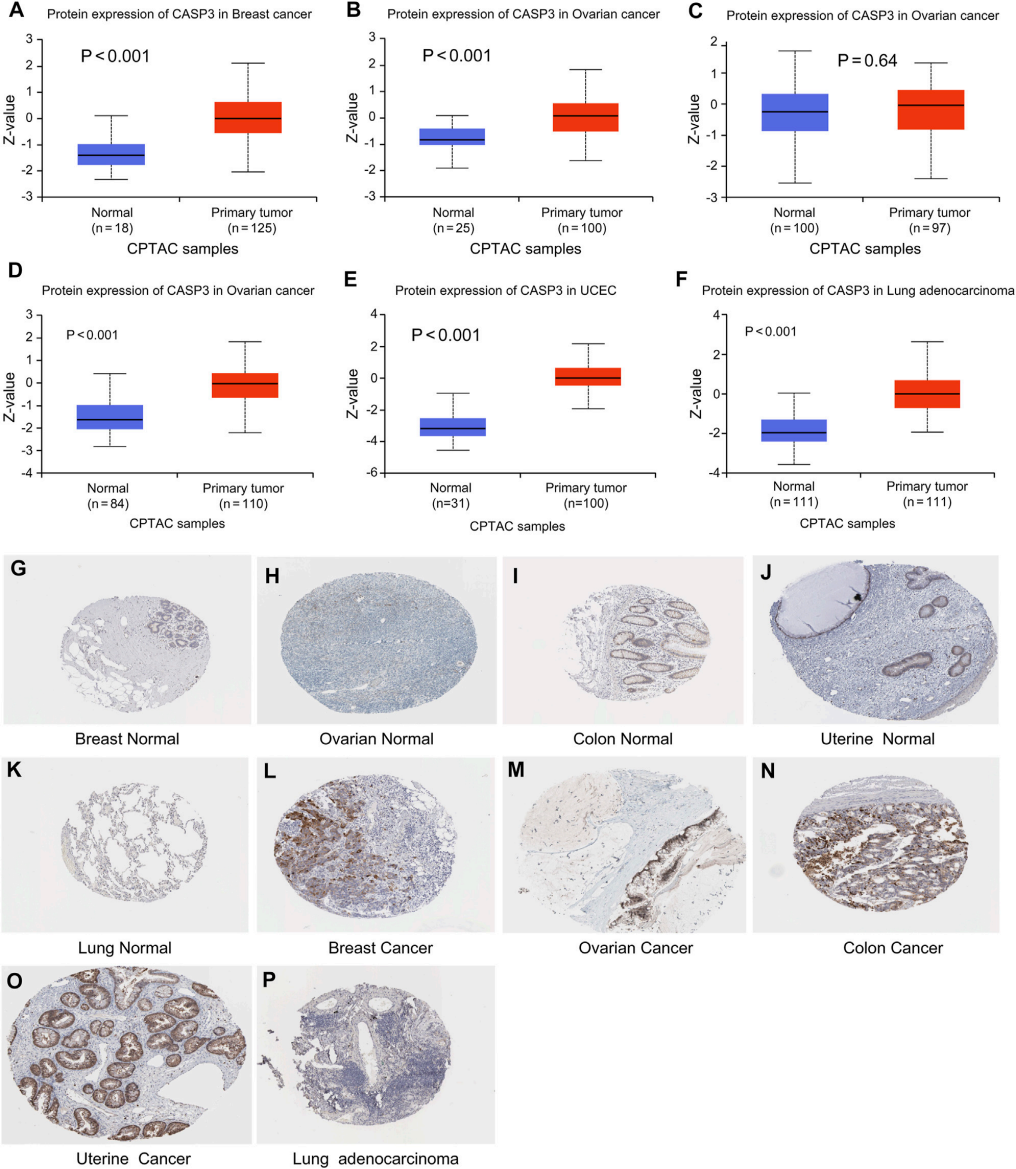
Levels of CASP3 in different tumours. Results based on CTPAC and HPA database **(A–F)** CASP3 is differentially expressed in breast cancer, ovarian cancer, colon cancer, clear cell RCC (renal cell carcinoma), UCEC and LUAD in tumour tissues and normal tissues in CTPAC database. **(G–P)** Expression of CASP3 in breast, ovarian, uterine and lung tissues was much higher than that in normal tissues.

Subsequently, to evaluate CASP3 expression at the protein level, we analysed the results of IHC staining in the HPA database and compared them with the levels of protein expression in six tumours obtained from the CPTAC database. The expression of CASP3 protein was significantly higher in all tumours, except in the colon (*p* < 0.001, Figures 2A–F). The remaining images indicate CASP3 IHC staining in normal tissues (Figures 2G–K) and in five tumours (Figures 2L–P). Unfortunately, no staining results have been reported for clear cell RCC. CASP3 IHC staining was low in normal tissues of the breast, uterus, ovary, and lung, and was moderate in normal tissues of the colon. Breast cancer and ovarian cancer tissues showed strong staining whereas colon cancer, UCEC, and LUAD tissues all showed moderate staining; with the exception of the colon, CASP3 protein expression in tumour tissues was significantly higher than that in normal tissues, which was consistent with the results obtained from the CPTAC database.

### Prognostic Analysis Data

**FIGURE 3:**
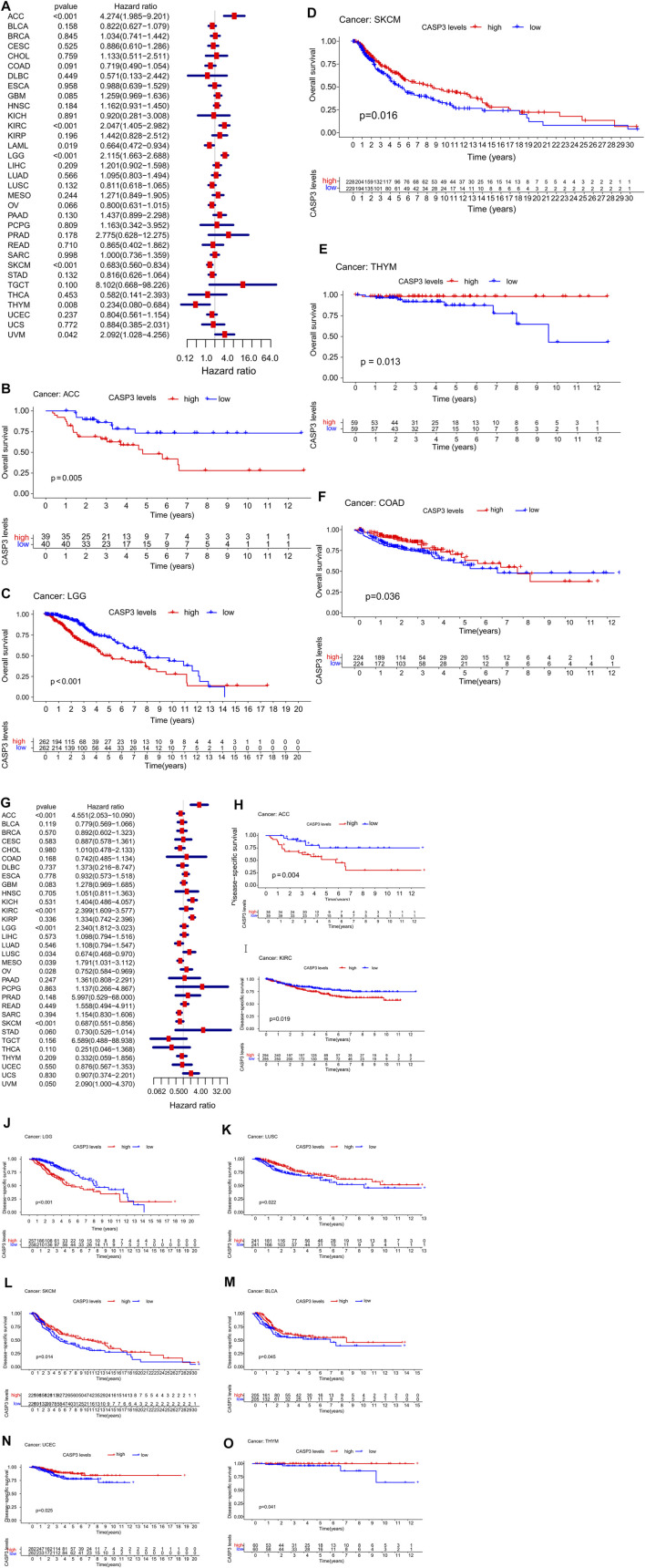
Relationship between *CASP3* expression and OS and DSS. **(A)** Expression of *CASP3* is associated with OS in 33 tumour types. **(B–F)** Kaplan-Meier survival analysis of *CASP3* expression and specific tumours’ OS. **(G)** Expression of *CASP3* and DSS related forest map of 33 tumour types. **(H–O)** Kaplan-Meier survival analysis of the relationship between *CASP3* expression and specific tumour DSS. Relationship between *CASP3* expression and OS and DSS (continuation of Figure 3).

Concerning prognosis, we performed survival association analysis for each tumour type, including OS, DSS, DFI, and PFI data. The Cox model of OS rate showed that *CASP3* expression was correlated with adrenocortical carcinoma (ACC, *p* < 0.001), KIRC (*p* < 0.001), acute myeloid leukaemia (LAML, *p* = 0.019), LGG (*p* < 0.001), SKCM (*p* < 0.001), thymoma (THYM, *p* = 0.008), and uveal melanoma (UVM, *p* < 0.042, Figure 3A). *CASP3* was identified as a high-risk gene in ACC, KIRC, LGG, and UVM, particularly in ACC, with a hazard ratio of 4.274, whereas it was a low-risk gene in the remaining tumours. Kaplan-Meier survival analysis also showed that high *CASP3* expression in ACC (*p* = 0.005, Figure 3B) and LGG (*p* < 0.001, Figure 3C) was associated with low OS, whereas high *CASP3* expression in SKCM (*p* = 0.016, Figure 3D), THYM (*p* = 0.013, Figure 3E), and COAD (*p* = 0.036, Figure 3F) was associated with better prognosis.

**FIGURE 4:**
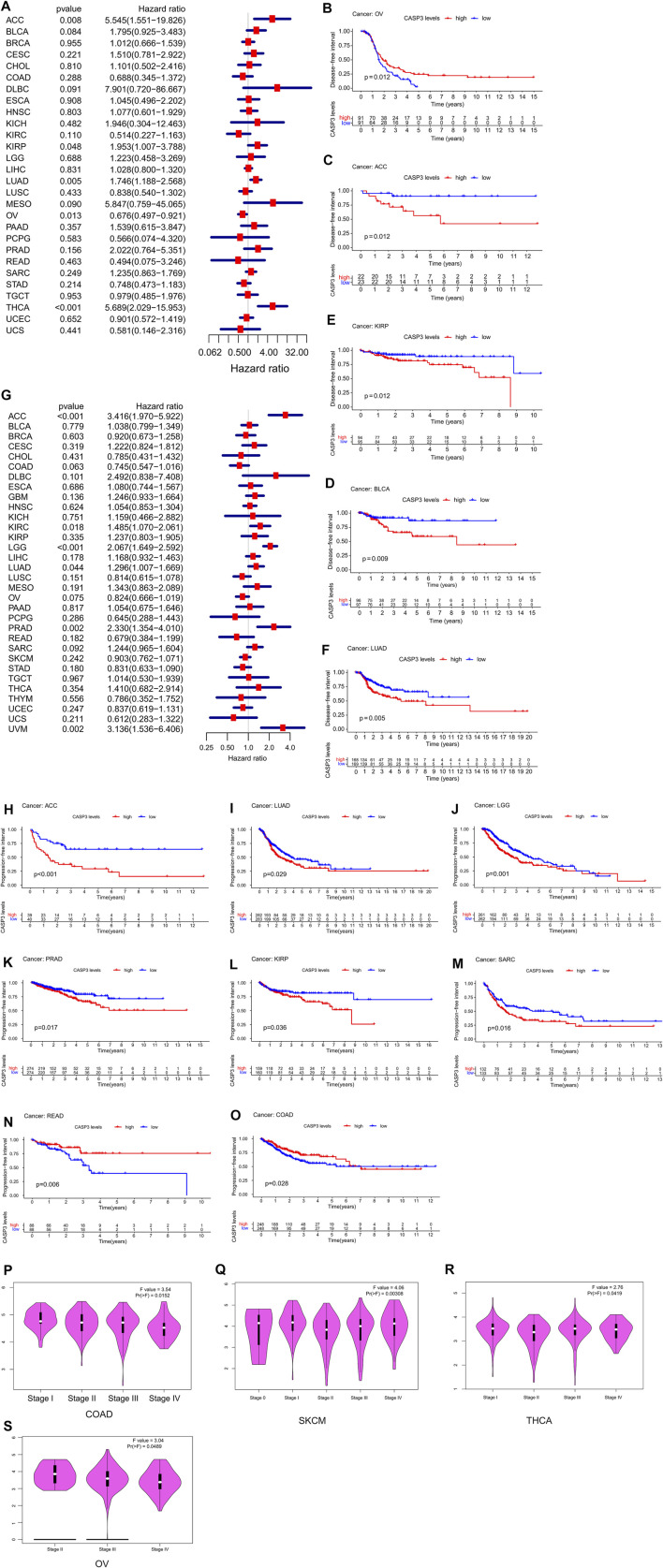
Relationship between *CASP3* expression and DFI, PFI, and different pathological stages. **(A)** Expression of *CASP3* is associated with DFI in 33 tumour types. **(B–F)** Kaplan-Meier survival analysis of the relationship between expression of *CASP3* and DFI in specific tumours. **(G)** Forest map of correlation between expression of *CASP3* and PFI in 33 tumour types. **(H–O)** Kaplan-Meier survival analysis of the relationship between the expression of *CASP3* and PFI in specific tumours. **(P–S)** Relationship between *CASP3* levels and different pathological stages of COAD, SKCM, THCA, OV. Relationship between *CASP3* expression and DFI, PFI, and different pathological stages (continuation of Figure 4).

Furthermore, in DSS, high *CASP3* expression in ACC (*p* < 0.001), KIRC (*p* < 0.001), LGG (*p* < 0.001), mesothelioma (MESO, *p* = 0.039), and UVM (*p* = 0.05) was associated with poor prognosis (Figure 3G). However, we observed opposing results in LUSC (*p* = 0.034), ovarian serous cystadenocarcinoma (OV, *p* = 0.028), and SKCM (*p* < 0.001). Kaplan-Meier survival analysis showed that high *CASP3* expression was not only associated with poor prognosis in ACC (*p* = 0.004, Figure 3H), KIRC (*p* = 0.019, Figure 3I), and LGG (*p* < 0.001, Figure 3J) but also with the prognosis of LUSC (*p* = 0.022, Figure 3K), SKCM (*p* = 0.014, Figure 3L), BLCA (*p* = 0.045, Figure 3M), UCEC (*p* = 0.025, Figure 3N), and THYM (*p* = 0.041, Figure 3O). In DFI (Figure 4A), in contrast to the CASP3-related good prognosis in OV (*p* = 0.013), *CASP3* overexpression was associated with poor prognosis in ACC (*p* = 0.008), KIRP (*p* = 0.048), LUAD (*p* = 0.005), and THCA (*p* < 0.001). The Kaplan-Meier survival analysis showed the same results for OV (*p* = 0.012, Figure 4B), with a good prognosis, whereas the prognoses of ACC (*p* = 0.012, Figure 4C), BLCA (*p* = 0.009, Figure 4D), KIRP (*p* = 0.012, Figure 4E), and LUAD (*p* = 0.005, Figure 4F) were poor. Finally, high *CASP3* expression was associated with low PFI in ACC (*p* < 0.001), KIRC (*p* = 0.018), LGG (*p* < 0.001), LUAD (*p* = 0.044), prostate adenocarcinoma (PRAD; *p* = 0.002), and UVM (*p* = 0.002, Figure 4G). Kaplan-Meier survival analysis showed that the high *CASP3* expression was associated with poor prognosis in ACC (*p* < 0.001, Figure 4H), LUAD (*p* = 0.029, Figure 4I), LGG (*p* = 0.001, Figure 4J), PRAD (*p* = 0.017, Figure 4K), and KIRP (*p* = 0.036, Figure 4L); high *CASP3* expression was also associated with poor prognosis in sarcoma (SARC; *p* = 0.016, Figure 4M) and good prognosis in READ (*p* = 0.006, Figure 4N), COAD (*p* = 0.028, Figure 4O). Further, we used the “Stage Plot” module of GEPIA2 to observe the relationship between *CASP3* expression and the pathological staging of tumours. We observed correlations between the expression of *CASP3* and COAD (Figure 4P), SKCM (Figure 4Q), OV (Figure 4R), and THCA (Figure 4S, *p* < 0.05). The remaining tumours are shown in Supplementary Figure S1.

### Genetic Methylation Analysis Data

**FIGURE 5:**
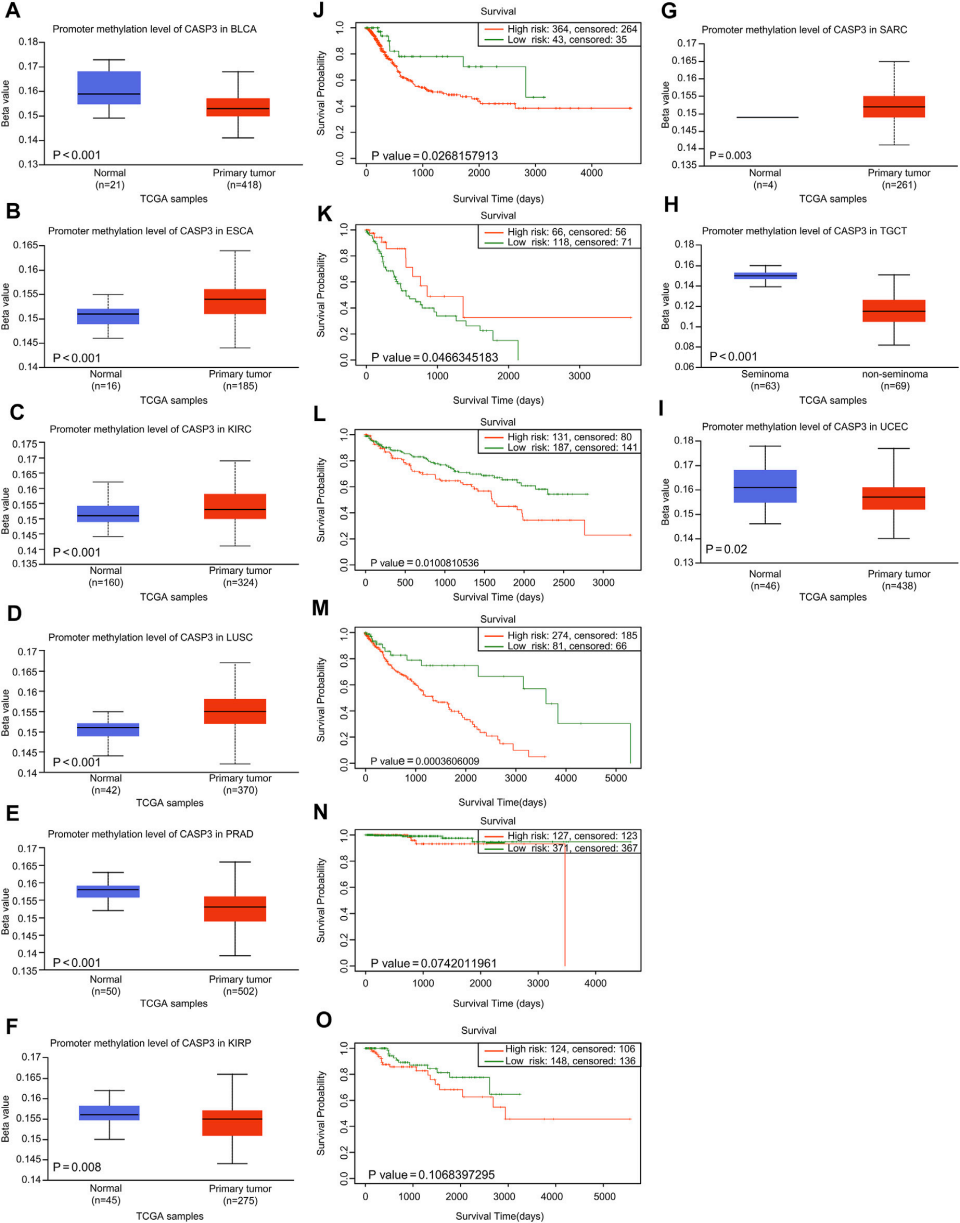
Relationship between *CASP3* expression and promoter methylation in different tumours. **(A–H)** Relationship between *CASP3* expression and promoter methylation in BLCA, ESCA, KIRC, LUSC, PRAD, SARC (Sarcoma), TGCT, UCEC. **(I–M)** Kaplan-Meier survival analysis of the relationship between *CASP3* promoter methylation level and OS in BLCA, ESCA, KIRC, LUSC, PRAD.

First, we analysed the differences in *CASP3* methylation levels in TCGA using the UALCAN database. In ESCA (Figure 5B), KIRC (Figure 5C), LUSC (Figure 5D), and SARC (Figure 5G), *CASP3* methylation levels were high, whereas in BLCA (Figure 5A), PRAD (Figure 5E), KIRP (Figure 5F), testicular germ cell tumours (TGCT; Figure 5H), and UCEC (Figure 5I), the reverse was true. We then used the “Single Case” plate of SurvivalMeth to analyse the relationship between *CASP3* methylation and tumour prognosis in TCGA database and obtain the Kaplan-Meier survival curve. Despite tumours that could not be analysed effectively because of lack of data, we observed that *CASP3* methylation levels only in ESCA (*p* = 0.047, Figure 5K) indicated a good prognosis, whereas in BLCA (*p* = 0.027, Figure 5J), KIRC (*p* = 0.010, Figure 5L), and LUSC (*p* < 0.001, Figure 5M), *CASP3* methylation levels were correlated with poor prognosis (*p* < 0.05). The levels of methylation in the other tumours and the associated prognoses are shown in Supplementary Figure S2.

### Gene Mutation Analysis Data

**FIGURE 6:**
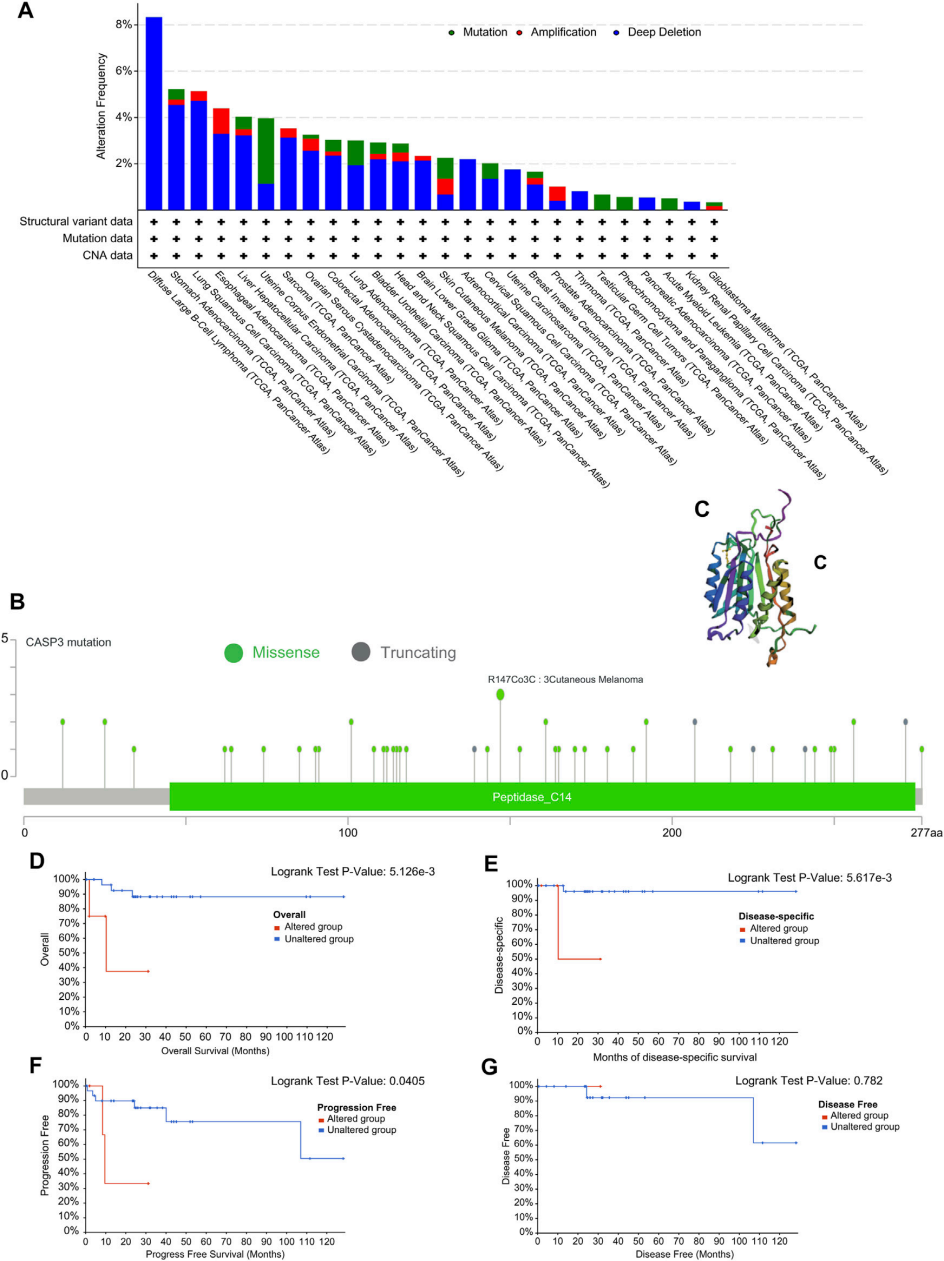
Mutation characteristics of *CASP3* gene in different tumours. **(A)** Types of mutations of *CASP3* in different tumours. **(B)** Frequency of *CASP3* mutation in different tumours. **(C)** Display the mutation site with the highest change frequency in the 3D structure of *CASP3*. **(D–G)** Relationship between *CASP3* mutation status and OS, DSS, DFI, PFI in DLBC by Kaplan-Meier survival analysis.

We observed genetic changes in *CASP3* in different TCGA tumours using cBioPortal. The highest frequency of *CASP3* mutations was 3% in UCEC (Figure 6A). In DLBC, “deep deletion” highlighted the full range of mutations (>8%). Notably, almost all tumours with mutations had a missing copy number of *CASP3*. All genetic loci of *CASP3* and the number of cases are shown in Figure 6B. The missense mutation of CASP3 was the main type of genetic change, accounting for more than 80% of cases. In all three cases, mutations at R147C were detected in SKCM. A 3D map of *CASP3* mutations at this site is shown in Figure 6C. In addition, we continued to explore the relationship between *CASP3* mutations and clinical survival. In DLBC, *CASP3* mutations resulted in poor OS (*p* = 0.005, Figure 6D), DSS (*p* = 0.006, Figure 6E), and PFI (*p* = 0.04, Figure 6F), therefore, it was chosen for the analysis as it had the most mutations. However, for DFI (*p* = 0.7, Figure 6G), which was not statistically significant because there was only one case of mutation data.

**FIGURE 7:**
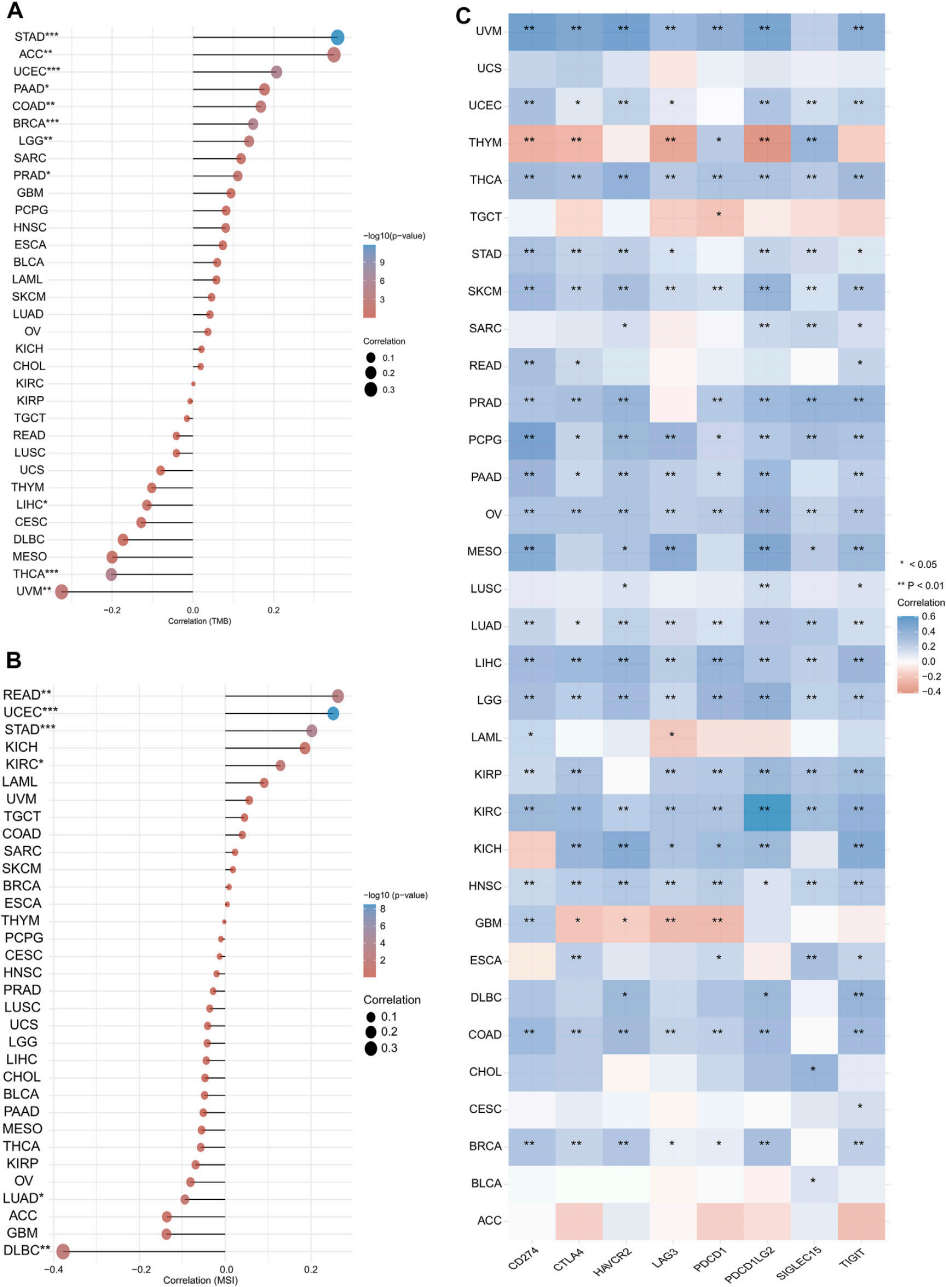
Expression of *CASP3* in relation to TMB, MSI and immunologic checkpoint. **(A)** the relationship between the expression of *CASP3* gene and TMB. *p < 0.05, **p < 0.01, ***p < 0.001. **(B)** Relationship between expression of *CASP3* and MSI. *p < 0.05, **p < 0.01, ***p < 0.001. **(C)** Correlation analysis between expression of *CASP3* and immunologic checkpoint-related genes in 33 kinds of tumours. *p < 0.05, **p < 0.01, ***p < 0.001.

We also analysed the association of *CASP3* expression with TMB and MSI in all tumours in TCGA database. In STAD (*p* < 0.001), UCEC (*p* < 0.001), BRCA (*p* < 0.001), ACC (*p* = 0.002), COAD (*p* = 0.002), LGG (*p* = 0.002), PRAD (*p* = 0.015), and pancreatic adenocarcinoma (PAAD, *p* = 0.025), *CASP3* expression was positively correlated with TMB, whereas was negatively correlated in UVM (*p* = 0.003), THCA (*p* < 0.001), and LIHC (*p* = 0.044, Figure 7A). In READ (*p* = 0.001), UCEC (*p* < 0.001), STAD (*p* < 0.001), and KIRC (*p* = 0.019), *CASP3* expression was positively correlated with MSI, but was negatively correlated with MSI in DLBC (*p* = 0.008) and LUAD (*p* = 0.03, Figure 7B).

### Tumour Microenvironment Analysis Data

**FIGURE 8:**
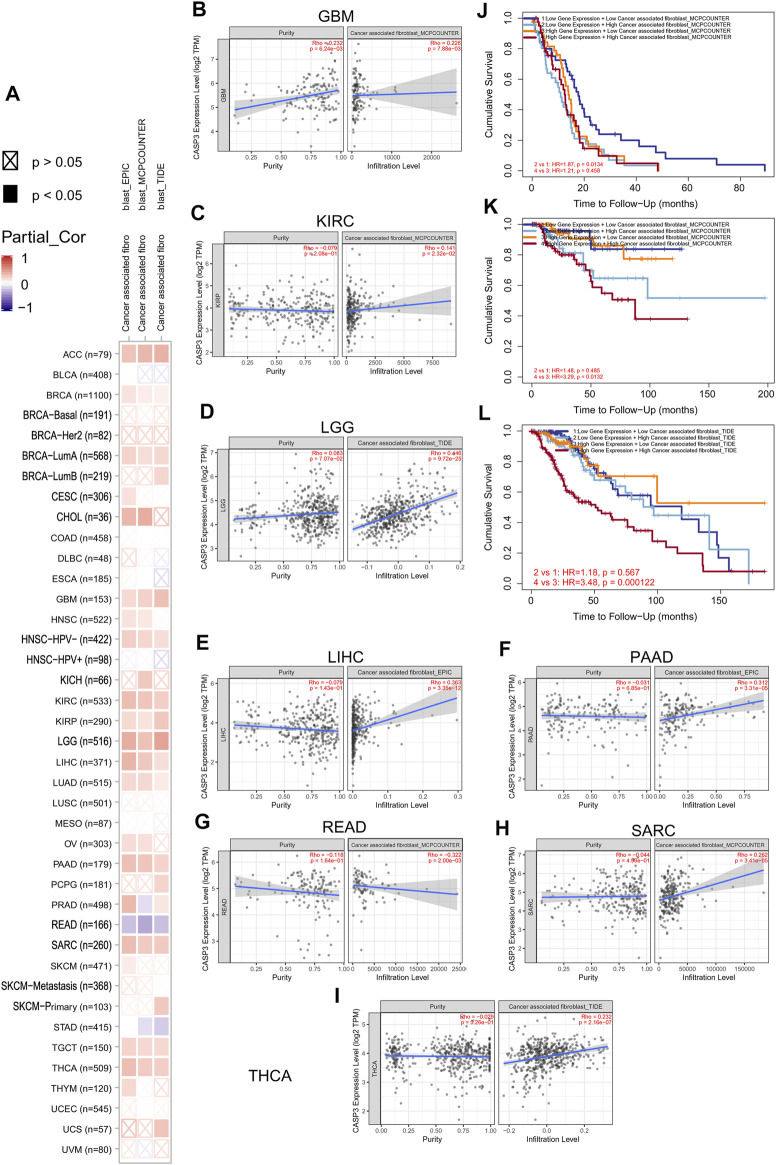
Relationship between the expression of *CASP3* and cancer-associated fibroblasts infiltration. **(A)** Relationship between expression of *CASP3* and infiltration of 33 kinds of cancer-associated fibroblasts based on three algorithms. **(B–I)** expression of *CASP3* in GBM, KIRC, LGG, LIHC, PAAD, READ, SARC, THCA and its correlation with fibroblasts infiltration. **(J–L)** Kaplan-Meier survival analysis of *CASP3* gene expression and fibroblasts infiltration in relation to OS of GBM, KIRC and LGG.

We first analysed the correlation between *CASP3* and eight immunologic checkpoint-related genes in all tumours. *CASP3* expression was negatively correlated (*p* < 0.05) with these immunologic checkpoint-related genes in all tumours, except in ACC, GBM, THYM, and TGCT (Figure 7C). Thereafter, the tumour microenvironment was analysed. Tide, MCP-counter, and EPIC algorithms were selected using TIMER2’s “Associated cancer fibroblast” module to analyse the relationship between *CASP3* expression and fibroblast infiltration in different TCGA tumours. *CASP3* expression was positively correlated with the invasion of fibroblasts in ACC, BRCA-lumA, HNSC-HPV, GBM, KIRC, KIRP, LGG, LIHC, LUAD, PAAD, SARC, TGCT, and THCA, but was negatively correlated with fibroblast infiltration in READ (Figure 8A); the relevant scatter plots are shown in Figures 8D–I. We further analysed the effects of *CASP3* expression and fibroblast infiltration on tumour prognosis. We chose the algorithm with obvious differences in prognosis; in turn, in GBM, KIRP, and LGG, poor prognosis was observed when fibroblasts were highly infiltrating (Figures 7J,K). The remaining relevant scatter plots are shown in Supplementary Figure S3.

**FIGURE 9:**
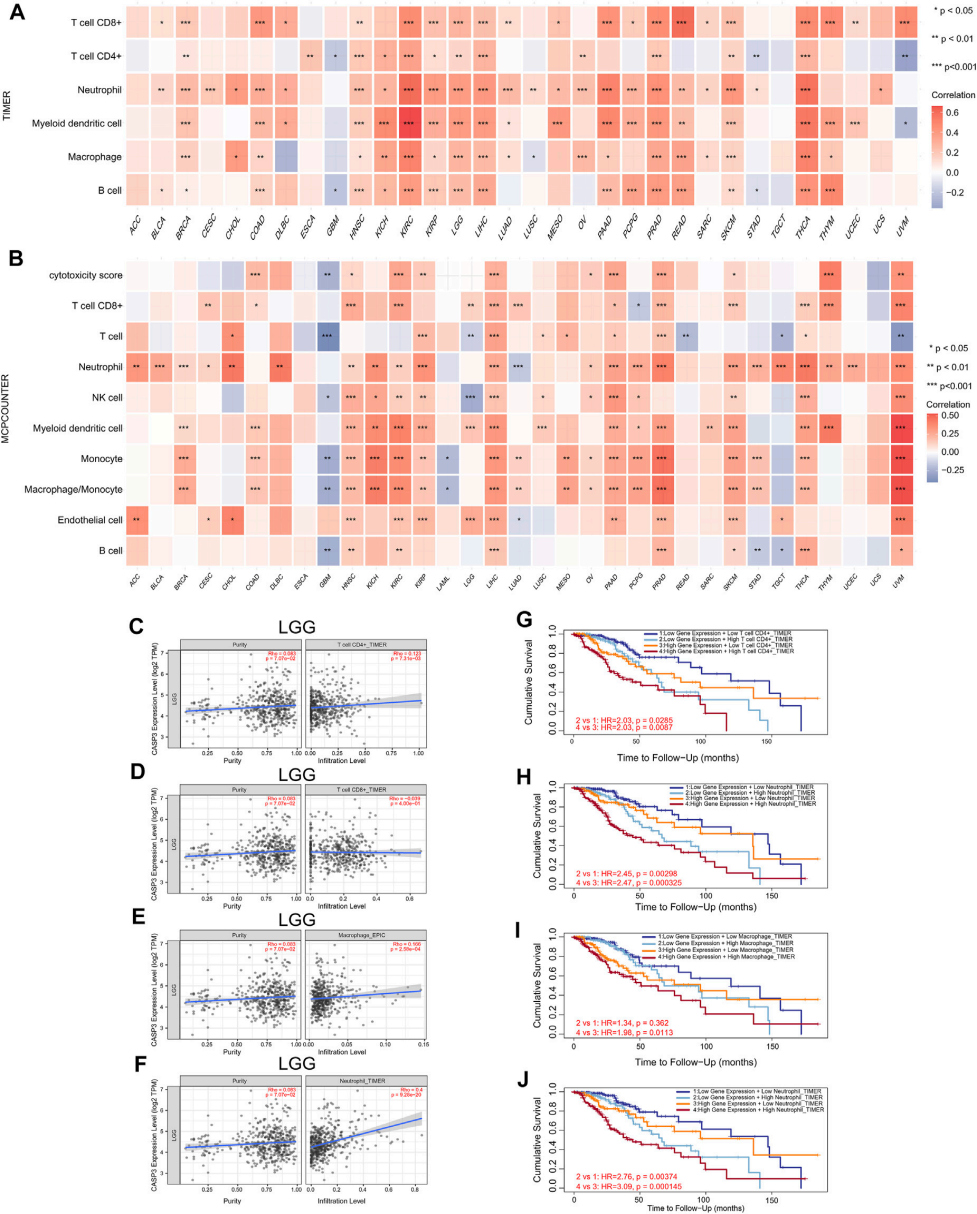
Relationship between the expression of *CASP3* and tumour immune cell infiltration. **(A)** Correlation between expression of *CASP3* based on TIMER algorithm and tumour immune cell infiltration. *p < 0.05, **p < 0.01, ***p < 0.001. **(B)** Correlation between the expression of *CASP3* based on MCP-counter algorithm and tumour immune cell infiltration. *p < 0.05, **p < 0.01, ***p < 0.001. **(C–F)** Relationship between the expression of *CASP3* in LGG and the infiltration of immune cells. **(G–J)** Kaplan-Meier survival analysis of *CASP3* expression and the relationship between immune infiltration and OS of LGG.

**FIGURE 10:**
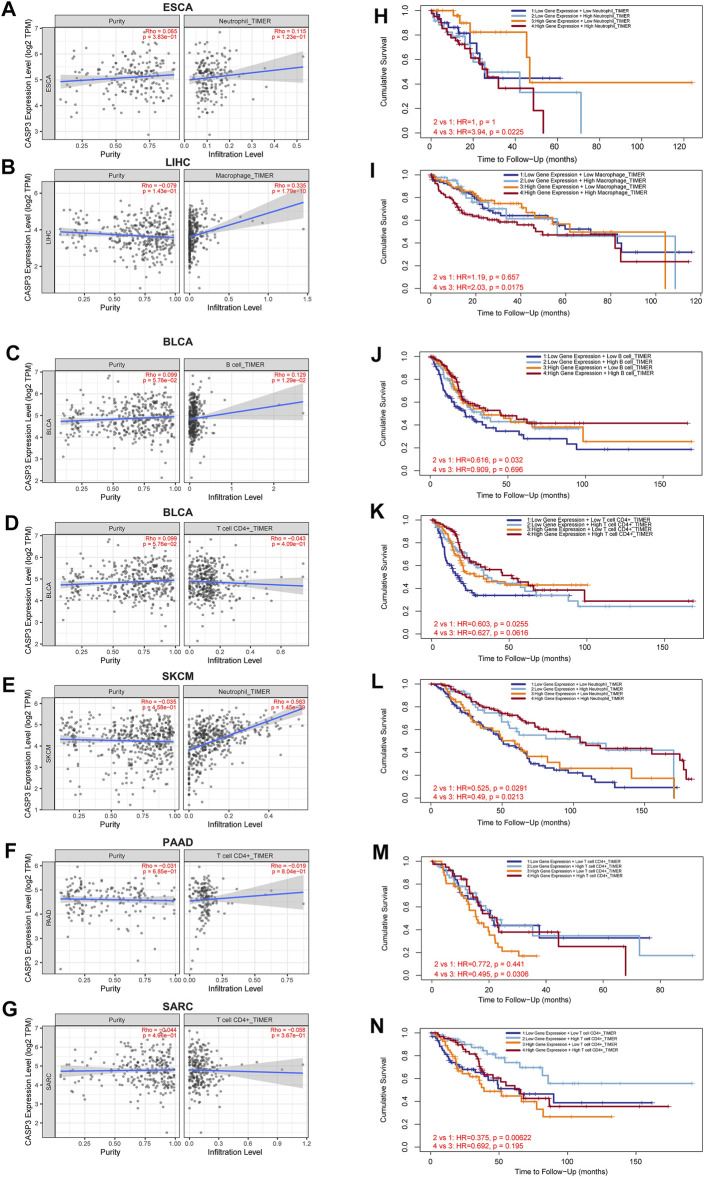
Relationship between *CASP3* expression and tumour immune cell infiltration. **(A–G)** the expression of *CASP3* in ESCA, LIHC, BLCA, skin cutaneous melanoma (SKCM), PAAD, SARC and its relationship with immune cell infiltration. **(H–N)** Kaplan-Meier survival analysis of *CASP3* expression and the relationship between immune infiltration and OS of ESCA, LIHC, BLCA, SKCM, PAAD, SARC.

Next, TIMER, xCell, MCP-counter, CIBERSORT, EPIC, and QUANTISEQ algorithms were used to investigate the potential relationship between the invasion level of different immune cells and *CASP3* expression in different cancer types in TCGA. Based on MCP-counter and TIMER algorithm data, we found that, almost all other tumours in the presence of *CASP3* expression was positively correlated with relevant immune cell infiltration in almost all tumours, with exception of ACC, GBM, and TGCT (Figures 9A,B). Based on several algorithms, we found that *CASP3* expression was closely related to the immune invasion of many cell types. We selected the TIMER algorithm and analysed the prognostic correlation. The scatter plots and Kaplan-Meier survival curve are shown in Figures 9C–J. Increased numbers of CD4^+^ T cells, CD8^+^ T cells, neutrophils, and macrophages in LGG were associated with poorer prognosis. High neutrophil infiltration in ESCA and high macrophage infiltration in LIHC were also associated with poor prognosis (Figures 10A–N). In BLCA, increased numbers of CD4^+^ T cells and B cells indicated an improved prognosis, and increased neutrophil infiltration in SKCM and increased CD4^+^ T cell infiltration in PAAD and SARC also predicted improved prognosis. The correlation scatter plots of the remaining tumours are shown in Supplementary Figure S4.

### Enrichment Analysis Data

**FIGURE 11:**
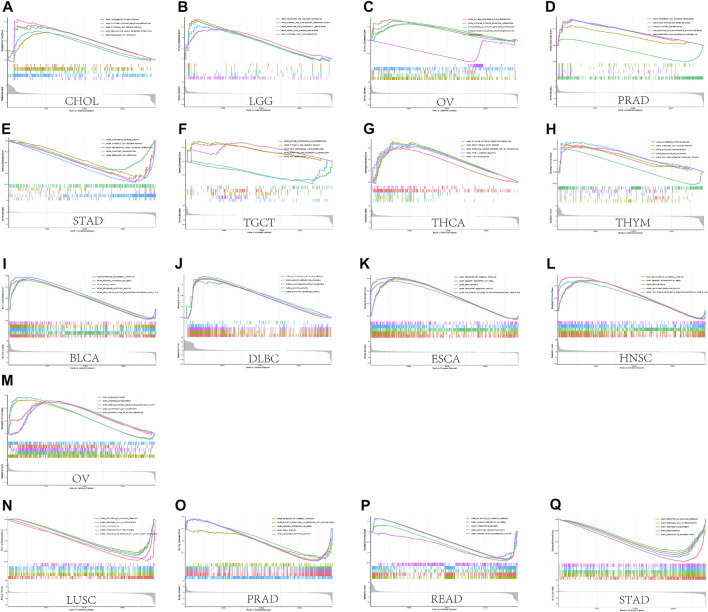
GSEA results. **(A)** Results of KEGG enrichment analysis of *CASP3* in multiple tumours. **(B)** GO enrichment analysis of *CASP3* in multiple tumours. Curves of different colours show different functions or pathways regulated in different cancers. Peaks on the upward curve indicate positive regulation and peaks on the downward curve indicate negative regulation.

We performed GSEA to investigate the biological significance of *CASP3* expression in different tumours from TCGA. The results based on KEGG and GO are shown in Figures 11A–Q. In KEGG enrichment analysis, *CASP3* expression in CHOL, LGG, OV, PRAD, THYM, and THCA was related to autophagy regulation, cytosolic DNA sensing pathway, cytokine receptor interaction, ascorbate and aldarate metabolism, neuroactive ligand receptor interaction, and porphyrin and chlorophyll metabolism, and mostly occurred through positive regulation, although it occurred through a negative regulation in STAD. These included related pathways that were positively regulated in most tumours, such as autophagy regulation and cytosolic DNA sensing pathway. Further, *CASP3* expression in OV and TGCT was positively regulated by antigen presentation and processing. In GO analysis, *CASP3* was most strongly associated with the detection of chemical stimulus pathways in almost all tumours, including the sensory perception of smell, olfactory receptor activity, and mRNA binding. These pathways were positively regulated in BLCA, ESCA, HNSC, and DLBC, and negatively regulated in STAD, LUSC, PRAD, and READ. However, the result of *CASP3* expression in OV in GO was the same as that in KEGG, indicating the involvement in B cell activation and immune response-regulating signalling pathways. *CASP3* also participates in the CCR chemokine receptor binding pathway and produces positive regulation in DLBC. The remaining tumour-associated GO enrichment analysis is shown in Supplementary Figure S5.

### *CASP3*-Related Gene Enrichment Analysis Data

**FIGURE 12:**
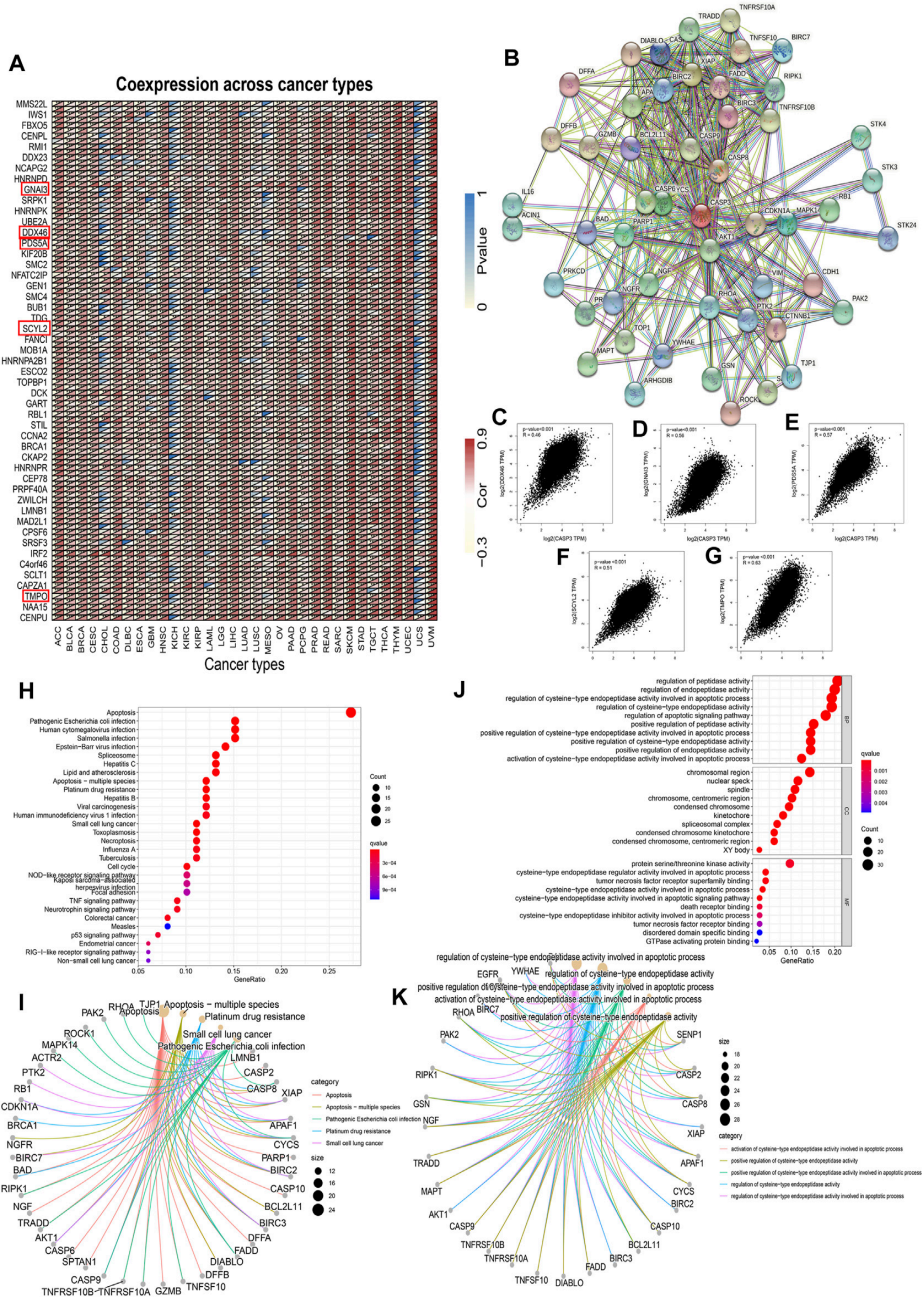
*CASP3*-related gene enrichment analysis. **(A)** Relationship between the *CASP3* and the first 100 genes in different tumours. The triangle in the upper left corner represents the *p*-value, and the triangle in the lower right corner represents the correlation coefficient. *p < 0.05, **p < 0.01, ***p < 0.001. **(B)** Interaction of CASP3 with the binding protein of the first 50. **(C–G)** Relationship between *CASP3* and the expression of selected genes (*DDX46*, *GNAI3*, *PDS5A*, *SCYL2*, *TMPO*) **(H,I)** based on *CASP3*-binding and correlated gene KEGG enrichment analysis results, and **(J,K)** based on *CASP3*-binding and correlated gene GO enrichment analysis results.

To further investigate the mechanism of *CASP3* in tumorigenesis and cancer progression, the first 100 genes related to *CASP3* expression were obtained from all tumour expression data in TCGA using GEPIA2. A heatmap of the correlation is shown in Figure 12A. In addition, we screened 50 CASP3-binding proteins from the STRING website, supported by experimental data, as shown in Figure 12B. The expression of *DDX46* (R = 0.46), *GNAI3* (R = 0.56), *PDS5A* (R = 0.57), *SCYL2* (R = 0.51), and *TMPO* (R = 0.63) were positively correlated with the *CASP3* levels (*p* < 0.001, Figures 12C–G). We then combined the two datasets for KEGG and GO enrichment analyses. According to the KEGG results (Figures 12H,I), in addition to apoptosis, *CASP3* may play a role in the tumour through infection and as a spliceosome. GO enrichment analysis data (Figures 12J,K) further showed that most of these genes affect cell biology by regulating the activities of apoptosis-related proteases, including peptidase and endopeptidase.

## Discussion

In recent years, an increasing number of studies have reported that *CASP3* is involved in cellular biological processes that cannot be defined simply by apoptosis; however, whether it is clinically relevant, and whether different tumours perform the same or different functions through the same molecular mechanism, remains unknown. A literature search failed to obtain any publications that analysed *CASP3* from the pan-cancer perspective. Therefore, we analysed the *CASP3* in 33 different tumours using the TCGA, CPTAC, and GTEx databases by investigating gene expression, genetic changes, gene methylation, and other molecular characteristics.

Our findings showed increased *CASP3* expression in 31 tumours in the TCGA database, 16 of which were statistically significant (*p* < 0.05), which was also demonstrated by protein expression analysis. In the meantime, we’re trying to find some clinical evidence that continues to support our view. Unfortunately, most studies have not studied the role of *CASP3* in related tumour tissues, the Caspase-3 protein has been used as a target in most of the researches.

However, Kaplan-Meier survival analysis of the CASP3 relationship showed a different result. For OS, we used GEPIA2 to analyse the statistical correlation between *CASP3* overexpression and overall survival outcomes, but our results differed from those obtained previously. We observed a significant correlation between *CASP3* overexpression and poor prognosis in ACC and LGG. The reason for this difference may be related to different data processing and analysis methods used. In addition, with the expression of *CASP3* in different tumours, the relationship between *CASP3* expression and survival may even appear to the contrary, which suggests that we need specific tumour analysis in the study of the relationship between *CASP3* expression and prognosis. In our analysis, we found that the expression of *CASP3* in HNSC and STAD was positively correlated with age and sex (*p* < 0.05, Supplementary Figure S6). This is consistent with previous findings (Huang et al., 2018) and may have important implications for guiding clinical immunotherapy regimens in the future.

DNA methylation is an important epigenetic mechanism that controls cell proliferation, apoptosis, differentiation, cell cycle, and transformation in eukaryotes, and many cancers are associated with promoter-specific hypermethylation (Morgan et al., 2018). Results from existing studies indicate that DNA methylation levels can be used as biomarkers for the early detection, diagnosis, and prognosis of cancer (Pan et al., 2018). We investigated, for the first time, the relationship between the methylation level of *CASP3* and different tumours, and its effect on clinical prognosis. Except for tumours without related methylation and prognosis data, significant results were obtained for clinical survival and prognosis. Therefore, we can conclude that the *CASP3* methylation level can be used as an independent prognostic factor for tumours. However, there are still few studies on the methylation of *CASP3*, which seems to be a promising direction in the future.

Recently, several studies have shown that *CASP3* expression often plays a role in tumours by participating in the pyroptosis process (Yu et al., 2019; Zhang et al., 2019), which is a type of programmed cell death in the form of inflammation, initially believed to be associated with innate immunity (Jorgensen and Miao, 2015). Subsequent studies have shown that pyroptosis in tumours can inhibit the proliferation and migration of tumour cells and, thus, affect prognosis (Fang et al., 2020). Therefore, we investigated the relationship between *CASP3* expression and immunity.

Both TMB and MSI are closely related to the tumour microenvironment and immunotherapy; hence, have recently received increasing attention. Recent studies have identified TMB as a biomarker for immunotherapy (Chan et al., 2019), as is considered to specifically affect immunotherapy outcomes by increasing the production of proteins recognised by the immune system in patients with high TMB. Immune cells are more likely to recognise and clear tumour cells with high TMB (Chen and Mellman, 2017; Chan et al., 2019). In patients with colorectal cancer, the sensitivity of MSI-high to immunologic checkpoint inhibitors (ICIs) is significantly higher than that of MSI-low (Lin et al., 2020). Herein, we found that *CASP3* expression in READ was closely related to MSI (*p* = 0.001). Studies have confirmed that the use of ICIs for treating tumours has become increasingly important in tumour immunotherapy (Darvin et al., 2018), and subsequent analysis of the correlation of immunologic checkpoints also revealed that *CASP3* plays a significant role in tumours. It is thus reasonable to conclude that tumours with high *CASP3* expression and positive association with TMB and MSI would be more sensitive to ICI therapy, which suggests an improved immunotherapy prognosis.

Tumour-infiltrating immune cells and cancer-associated fibroblasts in the tumour matrix, which are important components of the tumour microenvironment, are closely related to cancer occurrence, development, and metastasis (Lei et al., 2020), and targeted therapy of the tumour microenvironment has become the focus of the current research for anticancer therapies (Xiao and Yu, 2021). Our follow-up findings on the role of *CASP3* in immunologic invasion, especially in CD8^+^ T cells, are consistent with previous studies (Jaime-Sanchez et al., 2020; Morris et al., 2020). Our results indicate that *CASP3* expression is positively correlated with the immunologic invasion of most tumours and has wide tumour practicability. Tumour-infiltrating immune cells play an important role in inhibiting or promoting tumorigenesis and cancer progression (Lei et al., 2020). Moreover, our results indicate a relationship between different immune cell infiltration and prognosis in different tumours; for example, in LGG, high *CASP3* expression and high infiltration of most immune cells were associated with poor prognosis, which provides a new direction for future research on ICIs. However, in BLCA, the results were reversed, which is consistent with previous studies (Zhang et al., 2020). Furthermore, increased fibroblast infiltration is reported to support the growth, movement, and invasion of tumour cells, resulting in tumour development and metastasis (Kuzet and Gaggioli, 2016). To the best of our knowledge, our study is the first to demonstrate the relationship between *CASP3* expression and fibroblast infiltration in tumours and to reveal its association with poor prognosis. However, it is a pity that we try to further explore the relationship between cancer-associated fibroblast subpopulations and tumour, but we find that there is a lack of relevant research data.

The results of tumour enrichment and co-expression enrichment analyses were similar. In addition to apoptosis, the possible mechanisms of *CASP3* action may involve B cell activation, antigen presentation, immune responses, chemokine receptors, and inflammatory function, which is consistent with previous studies indicating that *CASP3* is a key protein in the regulation of tumour progression in addition to pyroptosis (Jiang et al., 2020). The enriched TNF and p53 signalling pathways in the KEGG bubble plot have also been studied to confirm that CASP3 plays an important role in the pyroptosis process in tumours (Wang et al., 2017; Wang et al., 2021).

## Conclusion

In summary, our pan-cancer analysis of *CASP3* showed a statistically significant association between gene expression and clinical prognosis, DNA methylation, gene mutations, tumour microenvironment, TMB, and MSI across multiple tumours, and possibly related molecular mechanisms, with carriable outcomes depending on the tumour type. Our study has some limitations, such as the unevaluated drug sensitivity of *CASP3* expression to ICIs and lacks experimental validation. However, to the best of our knowledge, this study is the first to explore the relationship between *CASP3* methylation in different tumours and the tumour microenvironment, along with the effect of these two factors on prognosis. These findings can help to further clarify the role of *CASP3* in tumorigenesis and development and provide a new reference for potential applications in immunotherapy.

## Data Availability

The datasets presented in this study can be found in online repositories. The names of the repository/repositories and accession number(s) can be found in the article/Supplementary Material.

## Supplementary Material

Supplementary Figure S1 Expression of *CASP3* is related to the different pathological stages of other tumours in the TCGA database.

Supplementary Figure S2 Relationship between the expression of *CASP3* in the remaining tumours in the TCGA database and the methylation of gene promoters and the related Kaplan-Meier survival analysis curve.

Supplementary Figure S3 Relationship between *CASP3* levels and the infiltration of other cancer-associated fibroblasts based on three algorithms.

Supplementary Figure S4 Based on the TIMER algorithm, the expression of *CASP3* is related to the infiltration of immune cells in other tumours.

Supplementary Figure S5 Expression of *CASP3* in the remaining tumours was based on the results of GO enrichment analysis.

Supplementary Figure S6 Expression of *CASP3* is related to clinically relevant traits such as age and gender of HNSC and STAD.
