# Supplementary figures and images for: A Systematic Pan-Cancer Analysis of CASP3 as a Potential Target for Immunotherapy
Source: Front Mol Biosci. 2022 Apr 29;9:776808. doi: 10.3389/fmolb.2022.776808 (PMC9106394; doi:10.3389/fmolb.2022.776808)

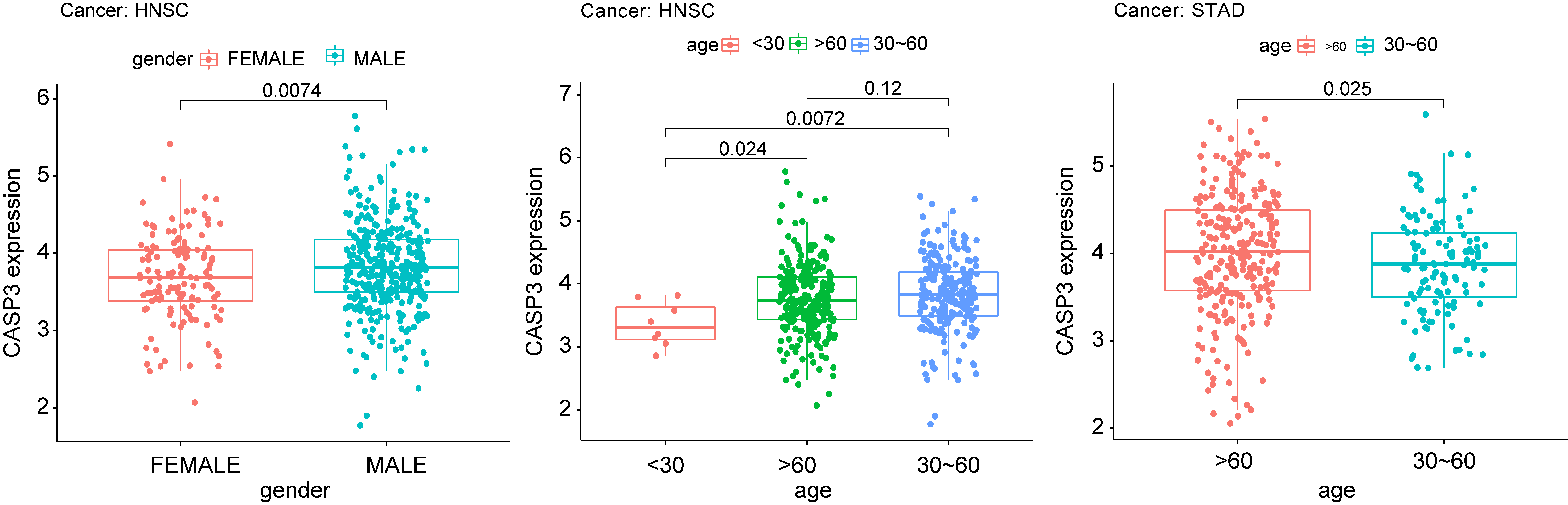

Supplement: Supplementary file 1 [file Image6.TIF]

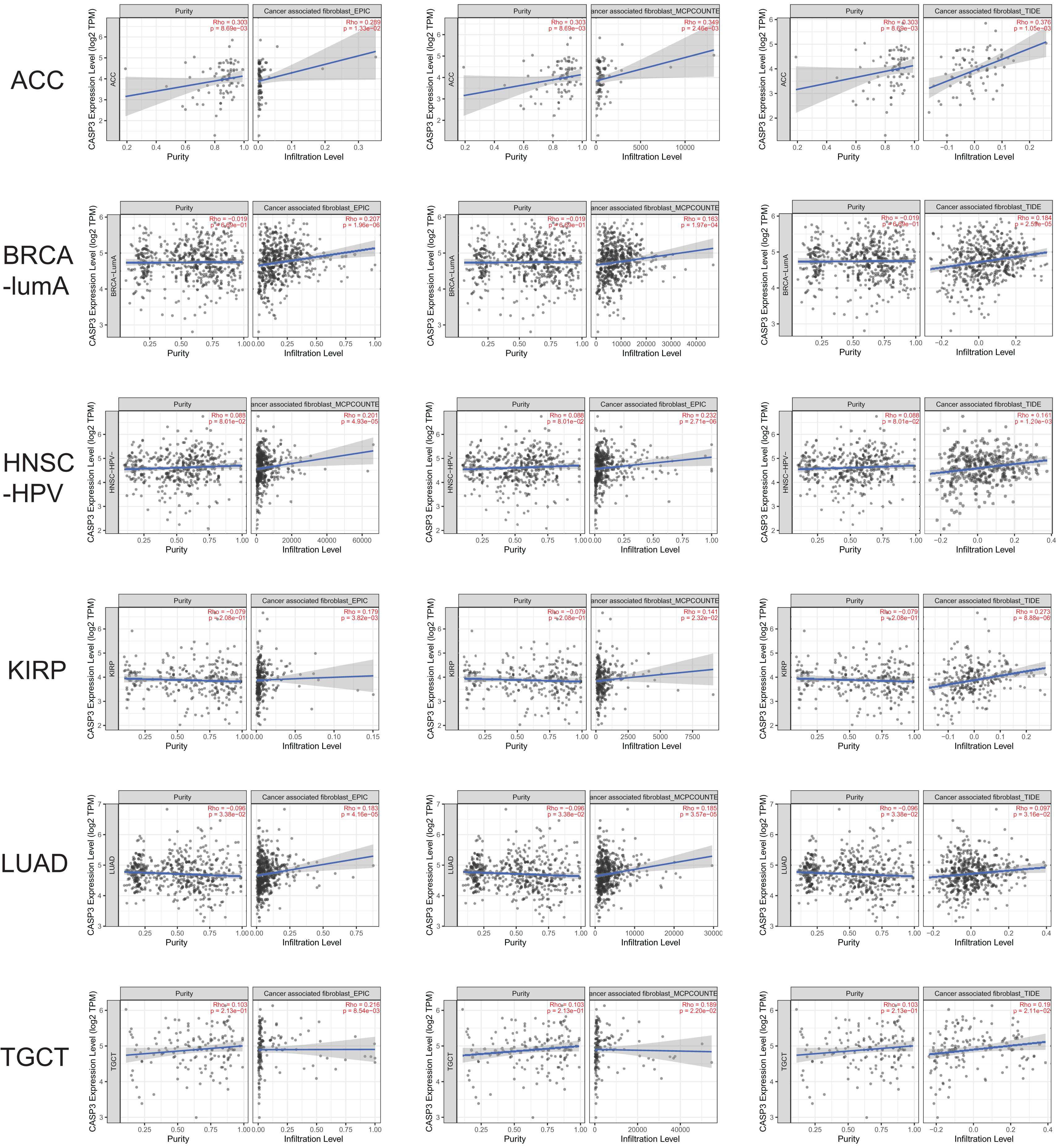

Supplement: Supplementary file 2 [file Image3.TIF]

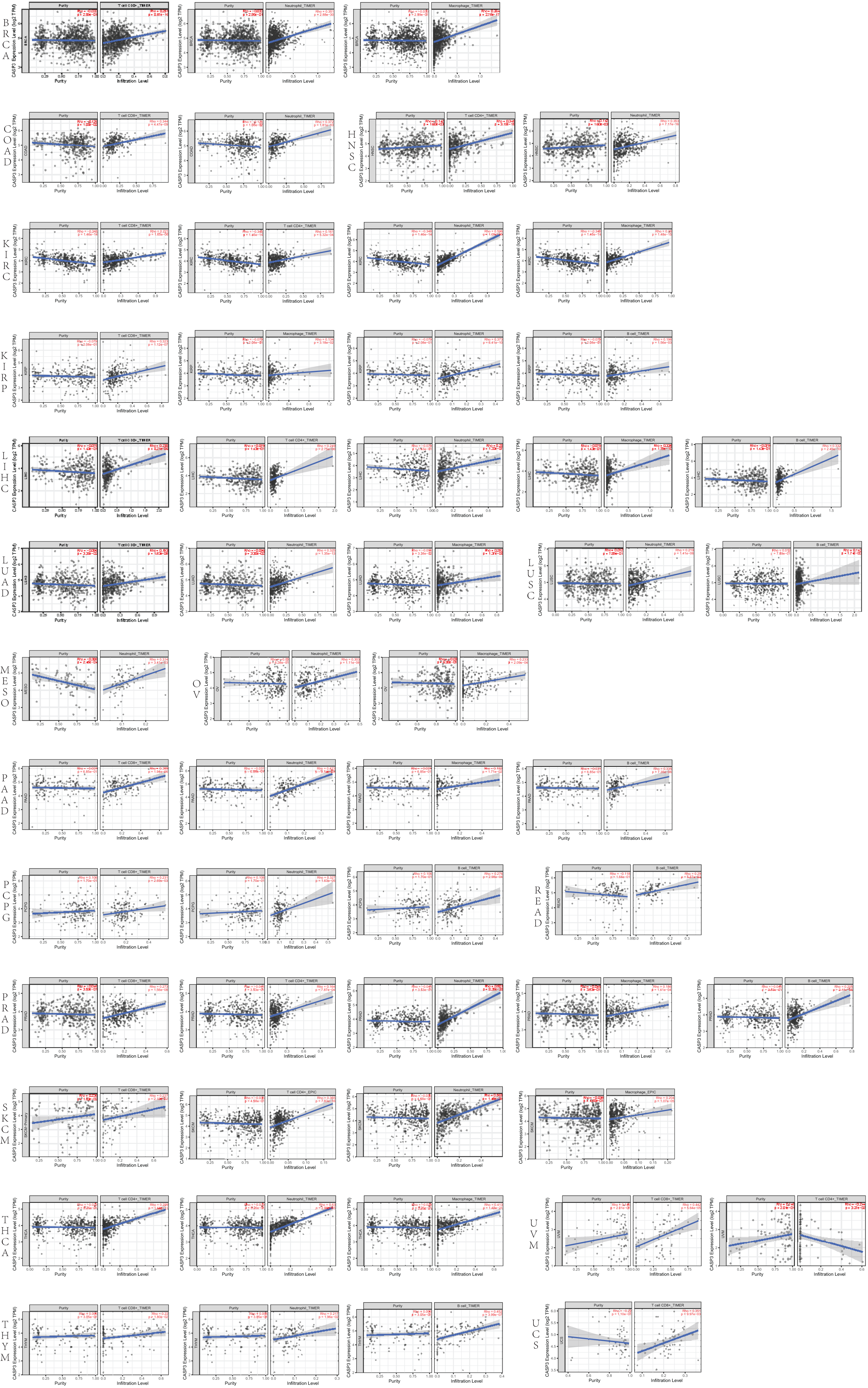

Supplement: Supplementary file 3 [file Image4.TIF]

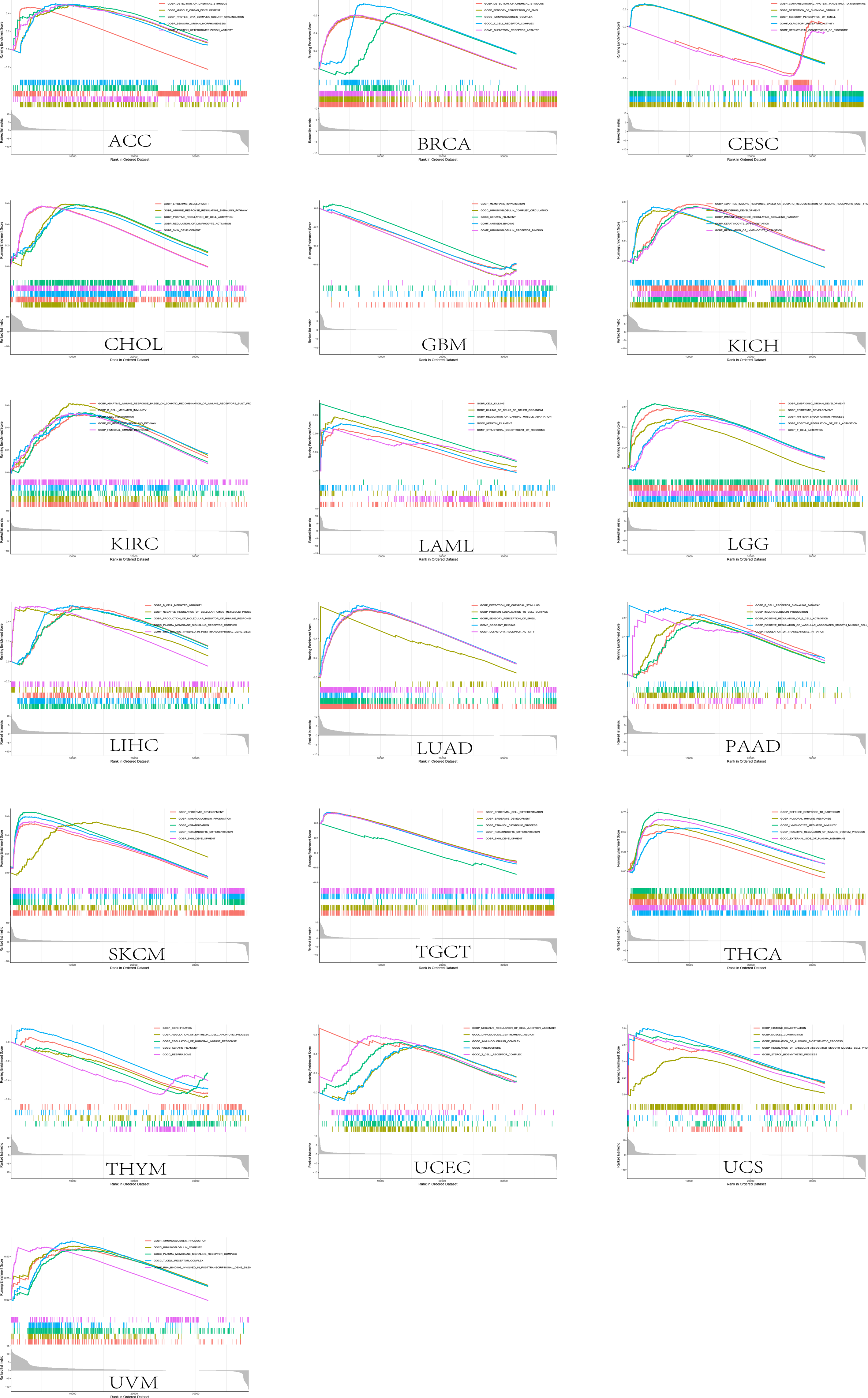

Supplement: Supplementary file 6 [file Image5.TIF]
